# Protocol for the isolation of human buccal cells for single-cell applications

**DOI:** 10.1016/j.xpro.2026.104601

**Published:** 2026-05-27

**Authors:** Supranee Buranapraditkun, Pongsawat Rodsaward, Natcha Chottawornsak, Pattarawat Thantiworasit, Jongkonnee Wongpiyabovorn, Hiroshi Chantaphakul

**Affiliations:** 1 Division of Allergy and Clinical Immunology, Department of Medicine, King Chulalongkorn Memorial Hospital, Faculty of Medicine, Chulalongkorn University, Thai Red Cross Society, Bangkok 10330, Thailand; 2 Division of Immunology, Department of Microbiology, Faculty of Medicine, Chulalongkorn University, Bangkok 10330, Thailand; 3 Division of Dermatology, Department of Medicine, Faculty of Medicine, Chulalongkorn University, Bangkok 10330, Thailand

**Keywords:** Health sciences, Clinical protocol, Immunology

## Abstract

Human buccal mucosa contains epithelial, stromal, and resident immune cell populations, but isolating these cells as a high-viability single-cell suspension is technically challenging. Here, we describe a gentle dissociation workflow for human buccal punch biopsies that combines controlled enzymatic digestion (collagenase and nuclease) with optimized speed, temperature, and incubation time to efficiently release heterogeneous cell populations while minimizing cell damage. The protocol reliably yields a heterogeneous single-cell suspension compatible with flow cytometry and single-cell RNA sequencing, with typical viabilities ≥80%.

## Highlights

• Instructions for buccal biopsy collection and rapid transport on ice
• Steps for tissue mincing and preparation for enzymatic digestion
• Controlled enzymatic digestion with collagenase and nuclease
• Procedures for filtration, washing, and counting for downstream single-cell analysis

## Abstract (editor highlights)

Publisher’s note: Undertaking any experimental protocol requires adherence to local institutional guidelines for laboratory safety and ethics.

## Graphical abstract

**Figure:**
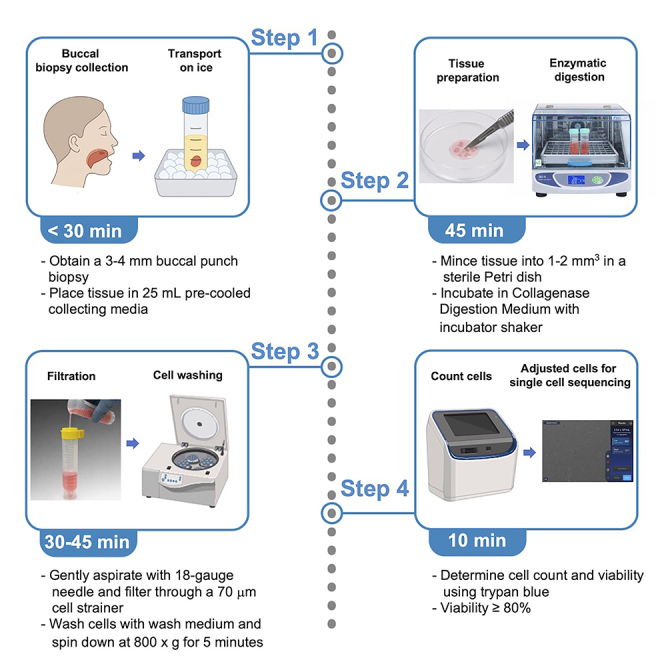

## Before you begin

This protocol describes the isolation of human buccal cells to generate a high-viability single-cell suspension suitable for downstream applications such as flow cytometry and single-cell sequencing.^1^^,^^2^^,^^3^^,^^4^^,^^5^ Buccal cells were selected to represent the local mucosal immune environment, in contrast to peripheral blood cells, which represent the systemic compartment. Refer to the key resources table and the materials and equipment sections for detailed reagent information and preparation steps.

1. Obtain human buccal biopsies under an Institutional Review Board (IRB)-approved clinical protocol, with informed consent from human subjects. Protocol is based on a single oral biopsy for downstream cellular and molecular analyses.
2. Place buccal tissue samples in 25 mL of pre-cooled (4°C) wash medium (RPMI 1640 supplemented with 1× Antibiotic-Antimycotic) in a sterile 50 mL conical tube, and transport to the laboratory on ice within 30 min of surgical collection to maintain cell viability.^1^^,^^6^
3. Ensure an adequate supply of stock solutions and reagents as described in materials and equipment section.
4. Perform all tissue processing and reagent preparation steps in a biosafety cabinet (Class II) to ensure sterility and operator safety.
5. Preheat an incubator shaker to 37^o^C before initiating enzymatic digestion.
6. Pre-cool a refrigerated centrifuge equipped with a 50 mL tube rotor to 4°C prior to use.
7. Prepare digestion buffer and tissue culture medium fresh on the day of the experiment.

### Innovation

This protocol presents an optimized workflow for isolating viable single cells from human buccal punch biopsies, a tissue type that is particularly susceptible to rapid degradation and mechanical stress. Compared with existing oral mucosal dissociation methods, this protocol introduces a rapid processing within 30 min of collection, a balanced enzymatic digestion using collagenase combined with nuclease to reduce DNA-mediated clumping, and gentle mechanical dissociation to minimize shear-induced cell death. The workflow is specifically optimized for small clinical biopsy samples (3–4 mm), enabling efficient recovery of heterogeneous cell populations including epithelial, stromal, and immune cells. Additionally, the protocol emphasizes temperature control and gentle handling to preserve cell integrity for single-cell transcriptomic applications. These improvements enhance reproducibility and compatibility with downstream single-cell technologies compared with conventional tissue dissociation protocols.

### Institutional permissions

All experimental procedures involving human samples were reviewed and approved by the Research Ethics Board, Faculty of Medicine, Chulalongkorn University (COA No. 0889/2025, IRB No. 0438/67).

### Participant criteria

Participants were adults aged 18–60 years with moderate to severe house dust mite–induced allergic rhinitis. Individuals were excluded if they had asthma, pre-bronchodilator FEV_1_ <70% of the predicted value, pregnancy, a history of allergen immunotherapy within the past 5 years, nasal surgery within the previous 3 months, or were receiving immunosuppressive medications. Participants were also excluded if they had malignancy, immunocompromising conditions, were current smokers, or regular alcohol consumption (>3 drinks per week). Additional exclusion criteria included oral inflammatory conditions (e.g., chronic oral ulcers, dental caries, or gingivitis), food allergy, and bleeding disorders, including the use of antiplatelet or anticoagulant medications.

## Key resources table

**Table:** 

REAGENT or RESOURCE	SOURCE	IDENTIFIER
**Biological samples**		

Human Buccal tissue (Age range: 18–56 years; both female and male)	Human Biopsy Tissue (Chula Skin OPD Clinic)	N/A

**Chemicals, peptides, and recombinant proteins**		

Benzonase® Nuclease	Merck	70664-3
Collagenase IV from Clostridium histolyticum	Sigma	C6885
RPMI Medium 1640	Gibco	11875093
Fetal Bovine Serum (FBS)	Gibco	26140079
Antibiotic-Antimycotic (100×)	Gibco	15240062
Trypan blue stain	Gibco	15250061

**Other**		

Scissors	–	N/A
Forceps	–	N/A
Scalpel Blade No. 21	Havels	N/A
70 micron cell strainer	Falcon	352350
Petri Dish	Falcon	351029
50 mL centrifuge tube	Biofil	CFT011500
15 mL centrifuge tube	Biofil	CFT011150
Refrigerated centrifuge (swinging-bucket or fixed-angle rotor)	Beckman Coulter	N/A
3 mL syringe	Nipro	N/A
10 mL syringe	Nipro	N/A
Hemocytometer	Neubauer, HBG	303212
Countess	Invitrogen	N/A
Auto pipette	Gilson	N/A
1 mL Serological pipette	Biofil	GSP010001
2 mL Serological pipette	Biofil	GSP010002
5 mL Serological pipette	Biofil	GSP010005
10 mL Serological pipette	Biofil	GSP010010
Shaker Incubator	N-Biotek	NB-205
Micro tube 2 mL	Sarstedt	72.694.006
MultiFit Tip 5-200 μL	Sorenson	SRS31930
MultiFit Tip 100-1000 μL	Sorenson	SRS10040

## Materials and equipment

**Table:** Collecting tissue media

Reagent	Final concentration	Amount
RPMI 1640	–	25 mL
Antibiotic-Antimycotic (100×)	–	250 μL

**Table:** Digestion media

Reagent	Final concentration	Amount
RPMI 1640	–	24.5 mL
Benzonase® Nuclease	20 U/mL	20 μL
Collagenase from Clostridium histolyticum	2 mg/mL	500 μL
Total	–	25 mL

Prepare within the experiment day and keep at RT

Use approximately 25 mL of digestion buffer for a 3–4 mm punch biopsy to ensure optimal performance.

**Table:** Counting media

Reagent	Final concentration	Amount
RPMI 1640	–	8.5 mL
FBS	15 %	1.5 mL

## Step-by-step method details

### Buccal biopsy collection

**Timing: 30 min for collection**

The purpose of this step is to collect tissue as quickly and aseptically as possible to preserve high cell viability for downstream applications.

1. Prepare a sterile 50 mL centrifuge tube containing 25 mL of ice-cold wash medium (RPMI 1640 + 1× Antibiotic-Antimycotic).
2. Collect buccal biopsies (3–4 mm punch) under local anesthesia by a trained clinician.
3. Immediately place the biopsy into the prepared wash medium on ice.
4. Transfer the samples to the laboratory within 15–30 min after collection for processing.

### Tissue preparation

**Timing: 30 min**

The step aims to mince the buccal tissue into small pieces suitable for enzymatic digestion. Although samples are transported on ice, dissection and subsequent procedures should be performed at 25^o^C in a biosafety cabinet (Class II).

5. Record the sample ID and time of tissue collection.
6. Prepare 25 mL of Collagenase Digestion Medium by adding 500 μl of collagenase IV and 20 μl of Benzonase immediately before use.^2^^,^^7^
7. Gently decant the wash medium from the biopsy tube, leaving approximately 1.5 mL to keep the tissue submerged.
8. Add 50 mL of wash medium to rinse the tissue, then gently remove it again, leaving approximately 1.5 mL to maintain the buccal tissue.
9. Transfer the buccal tissue to a sterile petri dish and record the buccal tissue weight (Figure 1A).

**Figure 1:**
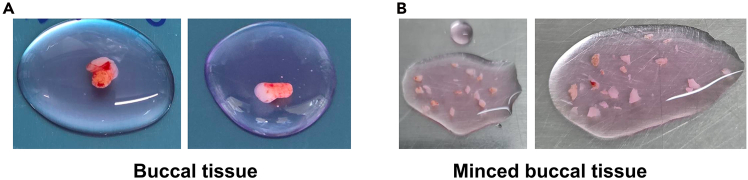
Buccal dissections (A) Buccal biopsy in Petri Dish containing tissue culture media. (B) Minced tissue prior to digestion step.
10. Using a sterile scalpel or scissors, mince the buccal tissue into small pieces (1-2 mm^3^) (Figure 1B).
11. Transfer the minced buccal tissue to a 50 mL tube containing 25 mL of the prepared Collagenase Digestion Medium.

### Enzymatic digestion

**Timing: 20 min**

This section describes the enzymatic digestion of minced buccal tissue at 37^o^C with shaking, followed by filtration and gentle mechanical dissociation to generate a single-cell suspension for downstream processing.

12. Seal the tube with Parafilm and place it on an orbital shaker or incubator shaker at 37^o^C, shaking at 140 rpm for 15 min to initiate digestion.
13. After incubation, allow the buccal tissue pieces to settle at the bottom of the tube.
14. Prepare a new 50 mL conical tube with a 70 μm Cell Strainer pre-wetted with 2 mL of wash medium.^2^^,^^8^
15. Carefully transfer the supernatant through the Cell Strainer, leaving approximately 5 mL in the original tube.
16. Using a 10 mL syringe with an 18-gauge needle, gently aspirate and expel the remaining buccal tissue suspension five times to further dissociate the cells.
17. Transfer this suspension through the same Cell Strainer, gentle crush remaining buccal tissue pieces using the syringe plunger.
18. Add wash medium to a final volume of 45 mL.

### Cell washing and counting

**Timing: 15–20 min**

This step describes centrifugation, resuspension, and viability assessment of cells using Trypan blue staining and followed by the adjustment of cell concentration for downstream applications or cryopreservation.

**Figure 2:**
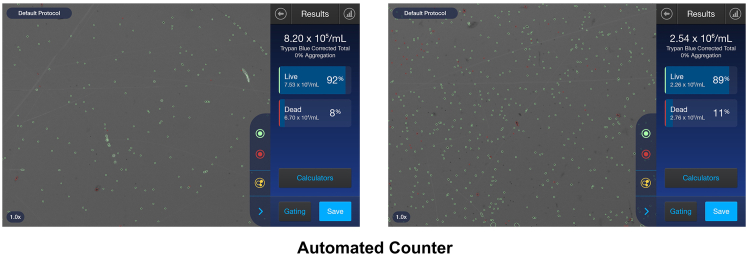
Measure cell count by automated cell counter and Trypan Blue staining
23. Adjust the cell concentration in tissue culture medium according to the requirements of downstream assays or prepare the cells for cryopreservation for later use.

***Note:*** Optimization of digestion conditions, including digestion medium composition, may be necessary depending on experimental goals.

19. Centrifuge the cell suspension at 800 × *g* for 5 min at 4^o^C.^1^^,^^7^
20. Carefully discard the supernatant and resuspend the cell pellet in 1 mL of R15 medium (RPMI 1640 + 15% FBS).
21. Take a 10 μl aliquot of the cell suspension and mix with 10 μl of Trypan Blue (1:1).
22. Count the total cell count, live cell count, dead cell count and viability using an automated cell counter (e.g., Countess) or a hemocytometer under a light microscope^5^^,^^9^^,^^10^ (Figure 2). A viability of ≥80% is recommended for downstream applications, such as single-cell RNA sequencing (e.g., droplet-based platforms typically require ∼5,000–20,000 cells with viability ≥85–90%) and flow cytometry (generally requiring ∼1 × 10^5^ to 1 × 10^6^ cells per sample, depending on the assay).

### Measure cell count using an automated counter

24. After completing cell isolation, assess total and viable cell numbers using an automated cell counter (e.g., Countess).

***Note:*** This step provides a quality control measure to evaluate the yield and reproducibility of buccal cell isolation (N = 14).

## Expected outcomes

**Figure 3:**
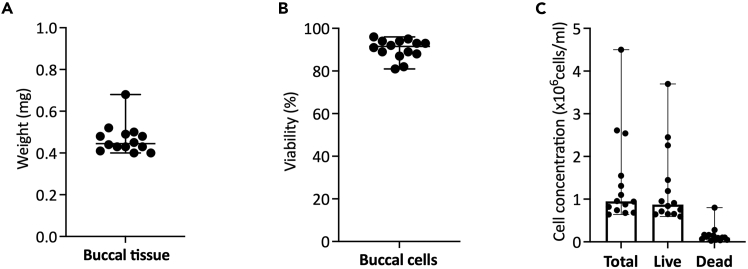
Human buccal tissue samples (A) Tissue weight, (B) Cell viability, and (C) Cell count data. The error bars represent the median with range.

This protocol yields a high-viability, heterogeneous single-cell suspension from human buccal tissue, suitable for downstream single-cell applications such as flow cytometry, transcriptomic profiling and single-cell RNA sequencing.^2^^,^^11^ The overall yield and viability depend on tissue integrity, digestion efficiency, and the time elapsed between biopsy collection and processing. Results from 14 human buccal samples showed tissue weight ranging from 0.40 to 0.68 g per 3-4 mm buccal biopsy (Figure 3A). Cell viability was ≥ 80% as assessed by Trypan Blue exclusion (Figure 3B), with yield of 0.5–3.7 × 10^6^ viable cells per 3–4 mm buccal biopsy (Figure 3C). The isolated cells comprised a heterogeneous population, including epithelial cells, fibroblasts, and immune cells. Morphologically, cells were predominantly round and intact, with minimal debris observed under light microscopy. When processed immediately after biopsy, the isolated buccal cells maintain high integrity and are compatible with single-cell library preparation systems.

## Limitations

This protocol has only been tested on buccal tissue and may need adjustments for use in other mucosal sites. The quantity and composition of buccal tissue can vary among individuals due to factors such as biopsy size, tissue density, and donor variability. All tissues were processed within 30 min of the biopsy; different results may occur if processing is delayed. Extended transport times or poor storage conditions can lead to cell death and RNA degradation, which may affect subsequent single-cell transcriptomic analyses. In addition, enzymatic digestion conditions require careful optimization and timing adjustments. Despite these challenges, following the recommended timing, temperature, and handling procedures will help ensure reproducible results and compatibility with single-cell applications.

## Troubleshooting

Here are some of the problems that might arise during the isolation of human buccal cells.

### Problem 1

Low cell yield due to incomplete tissue digestion or small biopsy size (Steps 5–11).

### Potential solution

Optimize digestion time by 10–20 min and adjust the mixing speed. Ensure fresh collagenase and Benzonase solutions are used. Mince tissue thoroughly before digestion.

### Problem 2

Low cell viability (<70%) due to delay between tissue collection and processing, over-digestion, or high shear stress (Steps 12–18).

### Potential solution

Process the tissue within 30 min of the biopsy. Reduce digestion time or agitation speed. Handle samples gently during pipetting and centrifugation.

### Problem 3

Presence of clumps or debris due to incomplete DNA digestion or inadequate filtering (Steps 12–18).

### Potential solution

Include Benzonase in the digestion buffer and filter the cell suspension through a 70 μm Cell Strainer before counting.

### Problem 4

Cell loss during washes due to incorrect centrifugation settings or pellet loss during aspiration (Steps 19–23).

### Potential solution

Centrifuge at 800 × *g* for 5 min at 4°C. Carefully aspirate the supernatant without disturbing the pellet and leave ∼100 μL of buffer above the pellet if needed.

### Problem 5

Low RNA quality in downstream assays due to cell degradation or prolonged processing time (Steps 19–23).

### Potential solution

Use freshly isolated cells or keep samples on ice during preparation. Use RNase-free reagents and consumables and proceed immediately to single-cell RNA sequencing (scRNA-seq) library preparation.

## Resource availability

### Lead contact

Further information and requests should be directed to the lead contact, Hiroshi Chantaphakul (hiroshi.c@chula.ac.th).

### Technical contact

Technical inquiries regarding the protocol should be directed to the technical contact, Supranee Buranapraditkun (supranee.b@chula.ac.th).

### Materials availability

This study did not generate new unique reagents.

### Data and code availability

This study did not generate datasets or code.

## Acknowledgments

We thank all study participants for their contributions to this research. We also acknowledge the Chula Medical Research Center (Chula MRC) for access to equipment and facilities. This work was supported by: (1) the Division of Allergy and Clinical Immunology, Department of Medicine, Faculty of Medicine, Chulalongkorn University, Bangkok, Thailand, and (2) the Division of Dermatology, Department of Medicine, 10.13039/100007693King Chulalongkorn Memorial Hospital, 10.13039/100032421Thai Red Cross Society, Bangkok, Thailand.

## Author contributions

S.B. developed and optimized the experimental procedure for the isolation of human buccal cells for single-cell applications and wrote the manuscript. P.R. contributed to writing, review, and editing of the manuscript. N.C. performed the buccal biopsy procedures. P.T. assisted with methodology development. J.W. conceived and designed the study. H.C. conceived and designed the study and contributed to writing, review, and editing of the manuscript.

## Declaration of interests

The authors declare no competing interests.
